# Osteogenesis enhancement by immobilized DOPA-BMP-2 in combination with ultrasonic stimulation†

**DOI:** 10.1039/d5ra02354h

**Published:** 2025-06-11

**Authors:** Kun Fang, Motoki Ueda, Xueli Ren, Yasuhiro Nakagawa, Yasutaka Anraku, Toshiyuki Ikoma, Yoshihiro Ito

**Affiliations:** a Nano Medical Engineering Laboratory, RIKEN Cluster for Pioneering Research Saitama 351-0198 Japan y-ito@riken.jp; b Graduate School of Material Science and Engineering, Institute of Science Tokyo Tokyo 152-8550 Japan; c Emergent Bioengineering Materials Research Team, RIKEN Center for Emergent Matter Science Saitama 351-0198 Japan

## Abstract

Bone morphogenetic protein-2 (BMP-2) plays a crucial role in regulating osteogenic differentiation and is widely used in tissue engineering. However, its clinical application is hindered by the high-dose administration of soluble BMP-2, thereby causing adverse effects and rapid degradation. Immobilizing BMP-2 on biomaterials offers an effective approach for achieving spatial and temporal control of growth factors while sustaining their bioactivity. In addition to growth factor signaling, mechanical forces regulate cellular behavior and interact with BMP-2 signaling pathways, potentially influencing cellular functions. In this study, we aim to investigate how the spatial presentation of BMP-2 affects osteogenic responses and its interaction with ultrasound stimulation. We observe that immobilized recombinant adhesive BMP-2 (DOPA-BMP-2) exhibits greater osteoinductive potential than its soluble counterpart, as indicated by the increased ALP activity and accelerated mineralization (93.9% *vs.* 77.5% on Day 14). Low-intensity pulsed ultrasound (LIPUS)—a mechanical stimulus—enhances osteogenic differentiation only when combined with immobilized DOPA-BMP-2, as evidenced by the upregulated ALP activity (71.7 *vs.* 58.1 mU μg_protein_^−1^) and larger mineralized area (90.4% *vs.* 72.6% on Day 7), whereas no significant effect is observed with soluble DOPA-BMP-2. Furthermore, cytoskeleton remodeling and focal adhesion formation are significantly enhanced exclusively under the combined treatment of immobilized DOPA-BMP-2 and LIPUS. These findings suggest that the enhanced osteogenic differentiation induced by immobilized BMP-2 and ultrasound may involve mechanotransduction pathways mediated by focal adhesion and cytoskeleton remodeling. This study supports the further development of BMP-2-functionalized biomaterials and biophysical therapy as a combined treatment for tissue engineering applications.

## Abstract 2

DOPA-BMP-2 immobilization combined with ultrasound enhances osteogenesis, providing an effective and biofriendly strategy for bone repair.

## Graphical abstract

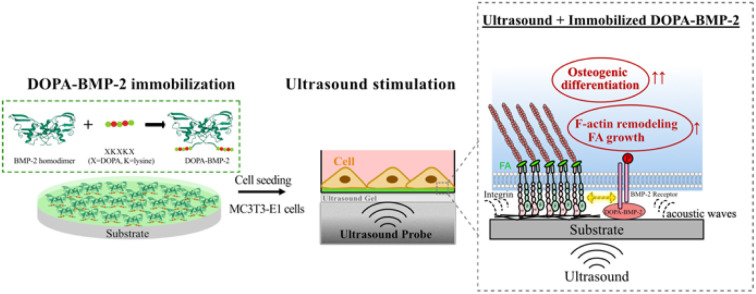

## 1 Introduction

Bone morphogenetic protein-2 (BMP-2) is crucial for cell migration and osteogenic differentiation during bone formation and has been approved by the U.S. Food and Drug Administration for clinical use. However, its clinical application is hindered by adverse effects associated with the high-dose administration of soluble BMP-2 for effective treatment.^1,2^ To address these challenges, BMP-2 immobilization on biomaterial surfaces has emerged as a promising tissue engineering strategy. The spatial presentation of BMP-2—whether on the dorsal or ventral side of cells—can result in distinct receptor cycling patterns and can affect both short- and long-term cellular responses.^3^ Numerous immobilization approaches have been explored, including physical adsorption, covalent coupling, and affinity-based interactions. Conventional physical binding of BMP-2 in matrices results in poor retention, offering only short-term effects.^4^ In contrast, covalent immobilization techniques, such as carbodiimide or thiol coupling, may chemically alter protein structure and restrict mobility, reducing the ligand-receptor signaling efficiency and thereby compromising bioactivity.^5^ Alterations in protein conformation or orientation during immobilization often result in bioactivity loss. Recent advances in site-directed covalent coupling, such as using engineered BMP-2 variants with non-canonical amino acids for click chemistry-based immobilization on collagen microspheres, have demonstrated enhanced *in vivo* bone formation.^6^ However, these strategies typically require chemical functionalization of the substrate surface to enable bio-orthogonal conjugation, which may limit their versatility and scalability. Affinity-based interactions, which exploit the natural binding between BMP-2 and extracellular matrix components or their functional domains (*e.g.*, heparin, collagen-binding motifs, or fibronectin fragments), offer a milder alternative that better preserves the conformation of BMP-2.^7–9^ Nevertheless, because these interactions depend on a limited number of binding sites, they may restrict the total amount of immobilized protein, potentially leading to compromised biological effects. Although these immobilization strategies have been explored, a major gap remains in balancing BMP-2 retention on biomaterials with the preservation of its bioactivity while ensuring mild reaction conditions and substrate-independent binding for practical use.

Mussel-inspired adhesion based on 3,4-dihydroxyphenylalanine (DOPA) offers a substrate-independent, rapid, and biofriendly strategy for surface functionalization; these characteristics make it highly suitable for immobilizing proteins on material surfaces.^10,11^ To harness this with growth factor immobilization, we previously developed a novel technique to recombine growth factors with mussel-inspired adhesive peptides through protein engineering and enzymatic post-modification.^12–14^ These adhesive peptides (DOPA-Lys-DOPA-Lys-DOPA) enable efficient immobilization under a mild aqueous conditions. Our previously developed DOPA-BMP-2 exhibited a high binding affinity for inorganic titanium and enhanced ligand–receptor interactions.^13^ However, its binding to organic materials, such as tissue-culturing polystyrene, and *in vitro* performance in facilitating osteogenesis remain unclear.

In addition to the biochemical signaling of growth factors, mechanical stimuli play a crucial role in regulating osteogenesis within the cellular microenvironment. Low-intensity pulsed ultrasound (LIPUS), a therapeutic mechanical force, stimulates bone regeneration in fresh fractures, nonunion, and delayed unions.^15–17^ It generates nanoscale motions in the tissue microenvironment, activating integrin-dependent mechanotransduction pathways.^18^ Previous studies have specifically focused on the combined effects of soluble BMP-2 and LIPUS on bone regeneration, with only limited combined effects reported.^19–22^ Because BMP-2-induced differentiation is closely associated with mechanotransduction pathways at the cell-extracellular matrix interface, the interaction between BMP-2 spatial presentation and ultrasound treatment requires further research.^23–25^ To the best of our knowledge, no studies have explored whether immobilized BMP-2, combined with therapeutic ultrasound, can enhance osteogenic differentiation.

**Fig. 1:**
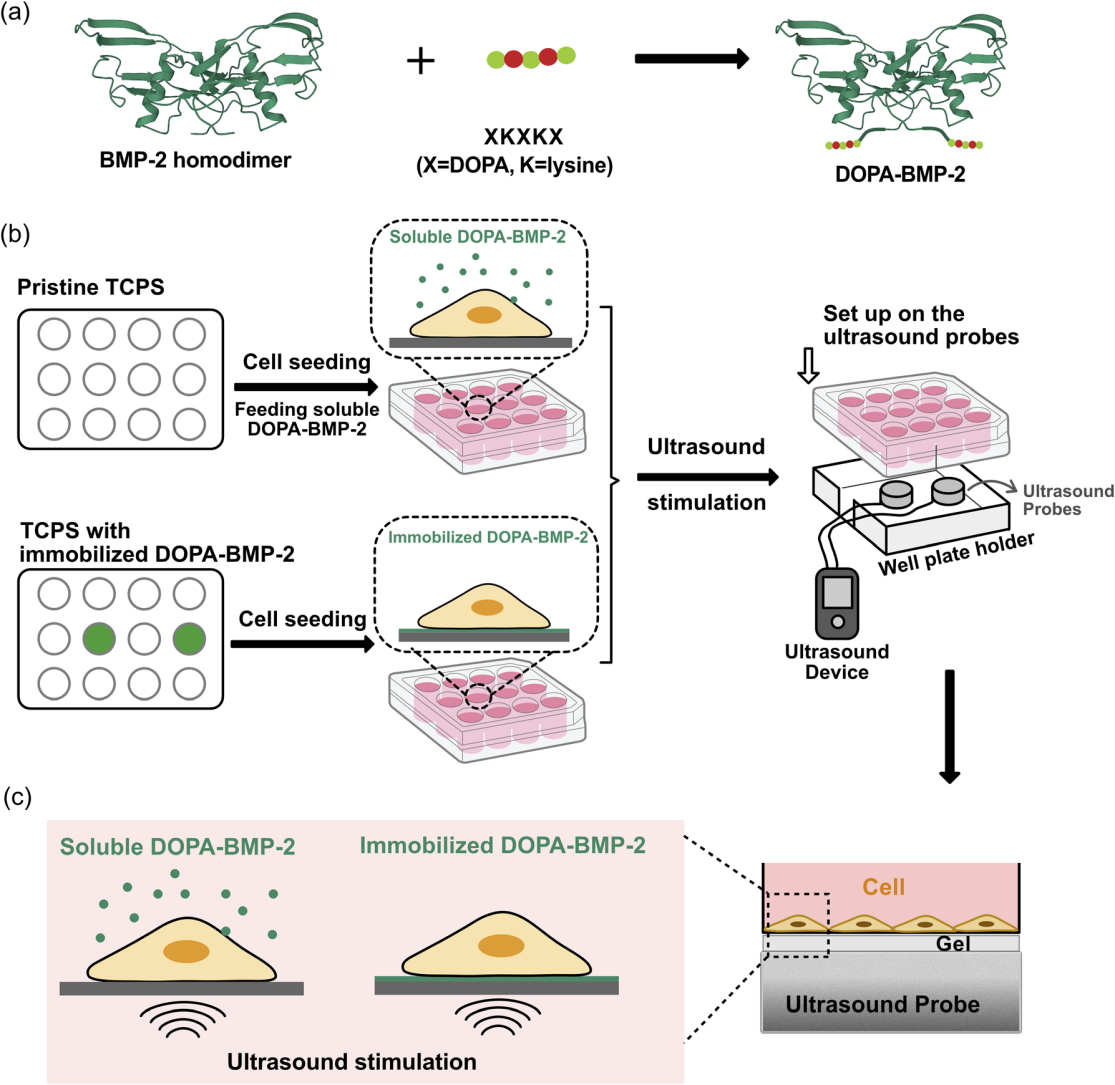
(a) Illustration of adhesive DOPA-BMP-2. (b) Workflow of the experiment procedure. (c) Detailed illustration of combining ultrasound stimulation with DOPA-BMP-2 in soluble or immobilized form for cell differentiation.

In this study, we aimed to investigate how the spatial presentation of DOPA-BMP-2 influences cellular responses and its interaction with therapeutic ultrasound. To this end, BMP-2 fused with DOPA-Lys-DOPA-Lys-DOPA polypeptides was prepared using an *Escherichia coli* overexpression system and was used in both soluble and immobilized forms, with ultrasound stimulation as the mechanical input (Fig. 1). The binding efficiency and surface properties were characterized, and the biological activity of DOPA-BMP-2-immobilized surfaces was compared to that of its soluble form. Additionally, the combined effects of LIPUS and BMP-2 signaling were assessed in terms of cellular mechanosensing and early- and late-stage osteogenic differentiation. These findings are expected to contribute to future clinical applications.

## 2 Experimental section

### 2.1 Preparation of BMP-2-modified surfaces

The recombinant BMP-2 variant containing DOPA-Lys-DOPA-Lys-DOPA polypeptides at the N-terminus was prepared using an *Escherichia coli* (*E.coli*) overexpression system and enzymatic post-modification, as illustrated in Fig. 1a, and carried out according to our published protocol.^13^ Briefly, the protein was expressed in *E. coli*, purified from inclusion bodies under denaturing conditions, refolded to obtain bioactive homodimers, and enzymatically hydroxylated to incorporate DOPA residues for surface adhesion. To immobilize DOPA-BMP-2 on polystyrene tissue culture plates (TCPS; Iwaki), the protein was diluted in Dulbecco's phosphate-buffered saline (D-PBS; Nacalai Tesque) to a predefined concentration. The solution was incubated on the TCPS surface with gentle shaking at room temperature for 30 min. Following incubation, the remaining solution was collected, and the surface was washed thrice with D-PBS to remove unbound proteins. The total amount of DOPA-BMP-2 remaining in the collected solution and washing buffer was quantified using a quantitative enzyme-linked immunosorbent assay (ELISA; PeproTech) with a standard calibration curve. The amount of DOPA-BMP-2 bound to the surfaces was calculated using eqn (1):

1 *A* = (*W*_1_ − *W*_2_)/*S*

where *A* is the surface binding amount (ng cm^−2^), *W*_1_ is the feeding amount (ng), and *W*_2_ is the remaining amount of DOPA-BMP-2 in solutions.

### 2.2 Surface properties

The hydrophilicity of DOPA-BMP-2-modified surfaces was assessed by measuring the water contact angle using a DropMaster Contact Angle Meter (Kyowa Interface Science) at room temperature. The surface morphologies of both pristine and BMP-2-bound TCPS were analyzed using a multimode atomic force microscopy (AFM; SHIMADZU) in tapping mode. Silicon cantilevers (Olympus) with a force constant of 1.7 N m^−1^ and resonance frequency of 70 kHz were used for the analysis.

### 2.3 Cell culture

The mouse calvaria-derived pre-osteoblast cell line (MC3T3-E1) was maintained in α-minimum essential medium (α-MEM; FUJIFILM Wako Pure Chemical Industries), supplemented with 10% fetal bovine serum (MP Biomedicals, LLC.) and 1% penicillin/streptomycin (Nacalai Tesque, Inc.) at 37 °C in a 5% CO_2_ atmosphere. Cells were passaged upon reaching 90% confluence. For the cell growth study, preosteoblasts were seeded at a density of 8 × 10^4^ cells per mL in a 96-well plate. For differentiation studies, pre-osteoblasts were seeded at a density of 5 × 10^4^ cells per mL and cultured for 3, 7, 14, and 21 days on BMP-2 immobilized surfaces or with soluble BMP-2. The α-MEM supplemented with 10 mM sodium β-glycerophosphate (Sigma-Aldrich), 50 μg mL^−1^l-ascorbic acid (FUJIFILM Wako Pure Chemical Industries), and 10 nM dexamethasone (MP Biomedicals, LLC.) was used for mineralization experiments. Fresh α-MEM and α-MEM with soluble DOPA-BMP-2 at the determined concentration were exchanged every 3 days for the immobilized and soluble BMP-2 groups, respectively.

### 2.4 Low-intensity pulsed ultrasound stimulation

To investigate the combined effect of mechanical and DOPA-BMP-2 stimulation on cell differentiation, we developed a workflow that integrates LIPUS application with cell culture treated with either soluble or immobilized DOPA-BMP-2 (Fig. 1b and c). One day after MC3T3-E1 cells were cultured with either soluble or immobilized DOPA-BMP-2, the well plate was placed onto ultrasound probes fixed within a custom-designed well plate holder to ensure stable and reproducible positioning during stimulation (Fig. 1b). Ultrasound was applied using an ultrasound gel on the probe surface, maintaining approximately 3 mm between the probe and bottom of the TCPS to maximize ultrasound penetration into the cell layer (Fig. 1c). The cell culture plates were positioned horizontally to ensure uniform ultrasound exposure. Ultrasound waves were delivered using a functional LIPUS device (Osteotron5; Ito Co., Ltd) with a pulse frequency of 100 Hz, ultrasound frequency of 1 MHz, and duty cycle of 20%.

To optimize the ultrasound conditions, we first screened ultrasound intensities of 30 or 60 mW cm^−2^ applied for 20 or 60 min per day, based on cell growth under ultrasound stimulation alone. For osteogenesis experiments, 30 mW cm^−2^ for 20 min daily was selected and applied during cell culture in an incubator at 37 °C with 5% CO_2_.

### 2.5 Cell growth under ultrasound treatment

Cell growth was assessed using Cell Counting Kit-8 (CCK-8; Dojindo). One day after ultrasound application, the cells were washed twice with D-PBS. Subsequently, 10 μL of CCK-8 solution was added to 100 μL of medium in each well, and the samples were incubated at 37 °C for 2 h. The relative cell growth was determined by measuring absorbance at 450 nm using a multimode microplate reader (PerkinElmer).

### 2.6 Alkaline phosphatase activity

Alkaline phosphatase (ALP) activity was measured using the LabAssay™ ALP kit (FUJIFILM Wako Pure Chemical Industries, Ltd) following the manufacturer's protocol. The cells were washed thrice with D-PBS, and lysis buffer (150 mM NaCl, 10 mM Tris–HCl, and 0.1% Triton X-100, pH 9.0) was added to each sample. The lysate was collected by scraping and then centrifuged (13 000 rpm, 15 min, 4 °C). The supernatant was used for a colorimetric reaction with *p*-nitrophenyl phosphate. After 30 min of incubation at 37 °C, the absorbance was measured at 405 nm using a multimode microplate reader. The ALP activity was normalized to the total protein concentration of each sample, which was determined using a NanoDrop spectrophotometer (Thermo Fisher Scientific).

### 2.7 Alizarin Red S staining

Alizarin Red S staining was performed to assess the mineralization of differentiated cells. After 7 or 14 days of culture, the cells were washed thrice with D-PBS and fixed with 4% paraformaldehyde phosphate (Nacalai Tesque). Following two washes with Milli-Q water, 2% Alizarin Red S solution (pH 4.2; Sigma-Aldrich) was added to each well, and samples were incubated at room temperature for 20 min. Subsequently, the cells were gently washed thrice with Milli-Q water to remove any unchelated stain. Stained samples were imaged, and the stained area percentage was analyzed based on a previously reported protocol.^26^

### 2.8 Immunofluorescent staining

MC3T3-E1 cells (4 × 10^4^ cells per mL) were seeded and cultured for 24 h in a humidified incubator at 37 °C with 5% CO_2_ before ultrasound stimulation. Staining was performed 1 h after stimulation because the cytoskeleton and FA approach mechanical homeostasis over time.^27^ Subsequently, the cells were washed twice with D-PBS and fixed with 4% paraformaldehyde phosphate at room temperature for 15 min. After fixation, the cells were washed and permeabilized with 0.1% Triton X-100 solution for 5 min. To prevent nonspecific binding, the samples were blocked with 1% bovine serum albumin for 1 h at room temperature. Subsequently, the samples were incubated with vinculin monoclonal antibody (1 : 200; FAK100, Sigma-Aldrich) for 1 h. After three washes, the cells were incubated with Alexa Fluor™ 488-conjugated goat anti-mouse IgG (1 : 500; Invitrogen) and tetramethylrhodamine isothiocyanate-conjugated phalloidin (TRITC-phalloidin; 1 : 1000; FAK100; Sigma-Aldrich) for 1 h at room temperature. Fluorescent images were captured using a fluorescence microscope (Olympus IX71, Tokyo, Japan).

### 2.9 Image analysis

The average number of focal adhesions per cell was quantified using Fiji (version 2.14.0/1.54f, ImageJ) from vinculin-stained images following an established protocol.^28,29^ Cytoskeletal actin anisotropy was analyzed using the Fiji plugin, FibrilTool.^30^

### 2.10 Statistical analysis

Data are presented as the mean value ± standard deviation. All tests were performed in three or more biological replicates. Statistical analyses were performed using one- or two-way analysis of variance, with Tukey's post-hoc test using GraphPad Prism version 9 (GraphPad Software, Inc., CA, USA).

## 3 Results and discussion

### 3.1 Surface binding of DOPA-BMP-2

**Fig. 2:**
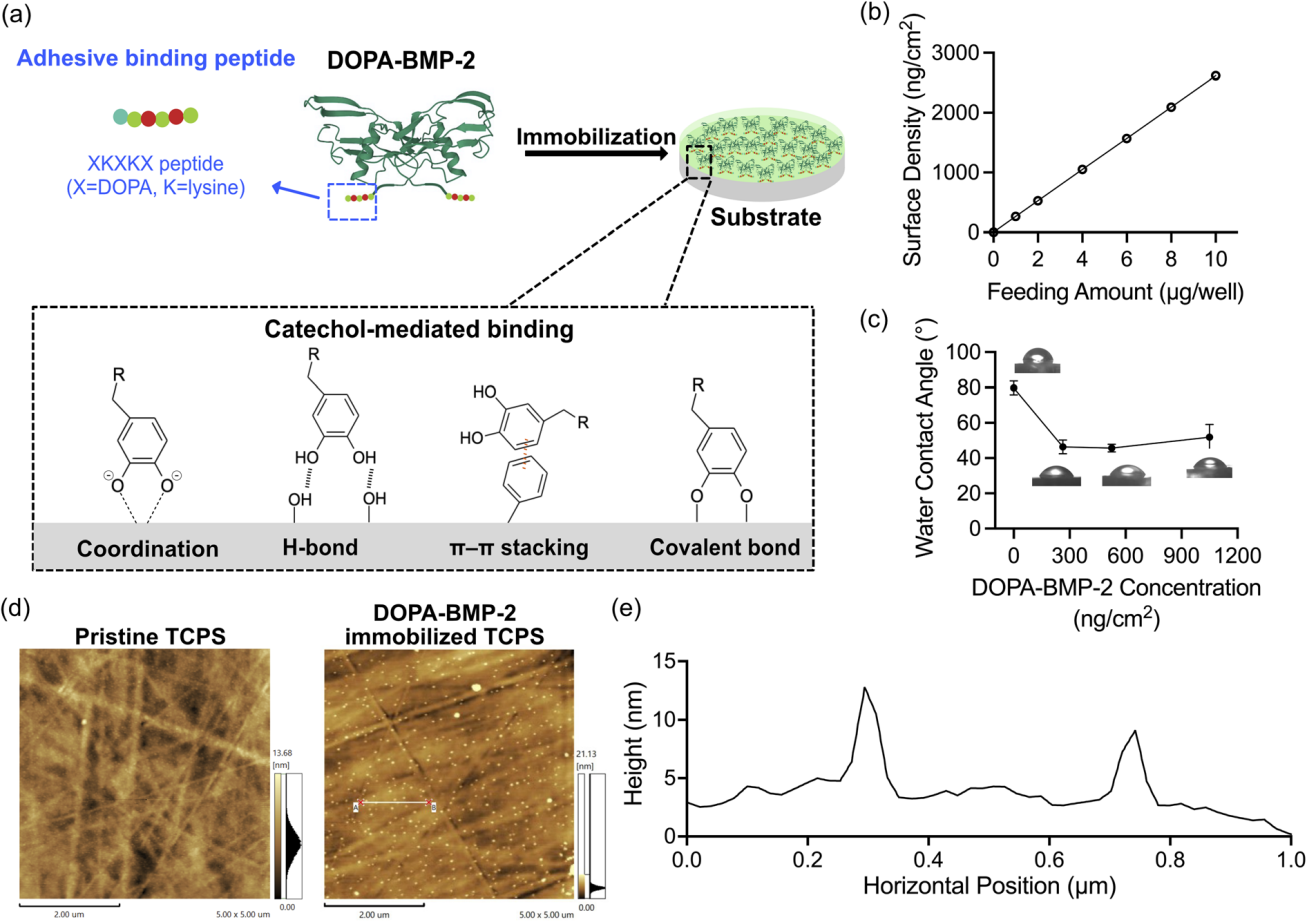
(a) Illustration of DOPA-BMP-2 binding and the mechanisms of catechol-mediated interactions with chemical groups on material surfaces. (b) The binding amount on TCPS surfaces in a 12-well plate. The error bar is not visible in the figure because of the high sensitivity of ELISA at the picogram scale. (c) Water contact angle values of pristine surface and surfaces bound with DOPA-BMP-2 at densities of 263 ng cm^−2^, 526 ng cm^−2^, and 1051 ng cm^−2^, respectively. (d) AFM images of pristine TCPS and TCPS immobilized by DOPA-BMP-2 at a density of 1051 ng cm^−2^. (e) Quantification of protein granule size by height measurement using AFM.

To enable surface immobilization of BMP-2 for subsequent cell experiments, a small pentapeptide (Tyr-Lys-Tyr-Lys-Tyr) was genetically introduced at the *N*-terminus of BMP-2 (Fig. S1a, ESI†), and the Tyr residues were enzymatically converted to DOPA using tyrosinase following protein refolding and purification. The successful extraction and purification of dimerized BMP-2 were confirmed by non-reducing sodium dodecyl sulfate-polyacrylamide gel electrophoresis based on its molecular weight (Fig. S1b, ESI†). The non-proteogenic amino acid DOPA exhibits strong binding affinities to various surfaces through catechol-mediated interactions (Fig. 2a).^10^ Our previous report demonstrated that DOPA-BMP-2 showed significantly higher binding affinity and surface retention on a titanium substrate than wild-type BMP-2 without DOPA modification.^13^ The binding amount of DOPA-BMP-2 on tissue culture polystyrene (TCPS) surfaces increased linearly with the feeding amount (Fig. 2b). Unlike previous multistep protein immobilization techniques, such as silanization^31^ and biotin/streptavidin-based immobilization,^32^ this binding method offers high stability under wet conditions^33^ and eliminates the need for additional surface treatment.

The water contact angle was measured to assess alterations in the wettability of growth factor-grafted surfaces (Fig. 2c). As the binding amount of DOPA-BMP-2 on TCPS increased (263, 526, and 1051 ng cm^−2^), the water contact angle reduced to approximately 45–50° across the three surfaces compared to 79.8° for pristine TCPS, indicating that the BMP-2-modified surfaces became more hydrophilic.

To further assess the effects of growth factor immobilization on the surface morphology, AFM phase scanning was performed (Fig. 2d). The pristine TCPS exhibited a characteristic fiber-like morphology, consistent with a previous study.^34^ Following DOPA-BMP-2 binding, the surface became less fibrous, with an increased number of protein granules. Quantitative analysis revealed that these granules were approximately 7.7 nm in size (Fig. 2e). For comparison, the crystal structure of BMP-2 dimer was approximately 7.2 nm, and its hydrodynamic size was 8.7 nm.^35^ These findings confirmed the uniform immobilization of recombinant adhesive DOPA-BMP-2 on the TCPS substrates.

### 3.2 Biological activity of BMP-2-immobilized surfaces

Following the successful immobilization of DOPA-BMP-2 onto TCPS, its biological activity was subsequently compared with that of the soluble form by evaluating both the short- and long-term osteogenic differentiation in MC3T3-E1 cells.

**Fig. 3:**
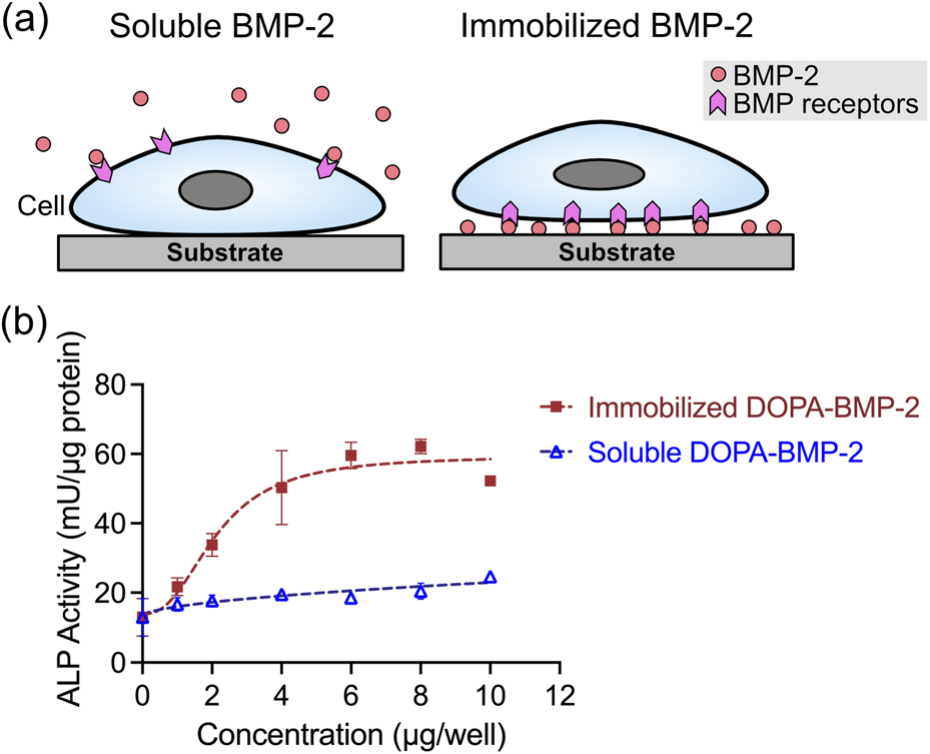
(a) Illustration of cells incubated with soluble or immobilized form of BMP-2. (b) ALP activity of MC3T3-E1 cells after 3 days culture with immobilized DOPA-BMP-2 or with soluble DOPA-BMP-2 in a 12-well plate.

Alkaline phosphatase (ALP), a primary marker of early osteogenic differentiation, was first quantified to assess the short-term response (Fig. 3). ALP expression was significantly higher in the immobilized BMP-2 group than that in the soluble group. On immobilized polystyrene surfaces, ALP activity increased in a concentration-dependent manner, plateauing at a concentration exceeding 6 μg per well. In contrast, soluble DOPA-BMP-2 induced a modest and sustained upregulation of ALP expression; however, it remained significantly lower than that observed in the BMP-2-immobilized group.

**Fig. 4:**
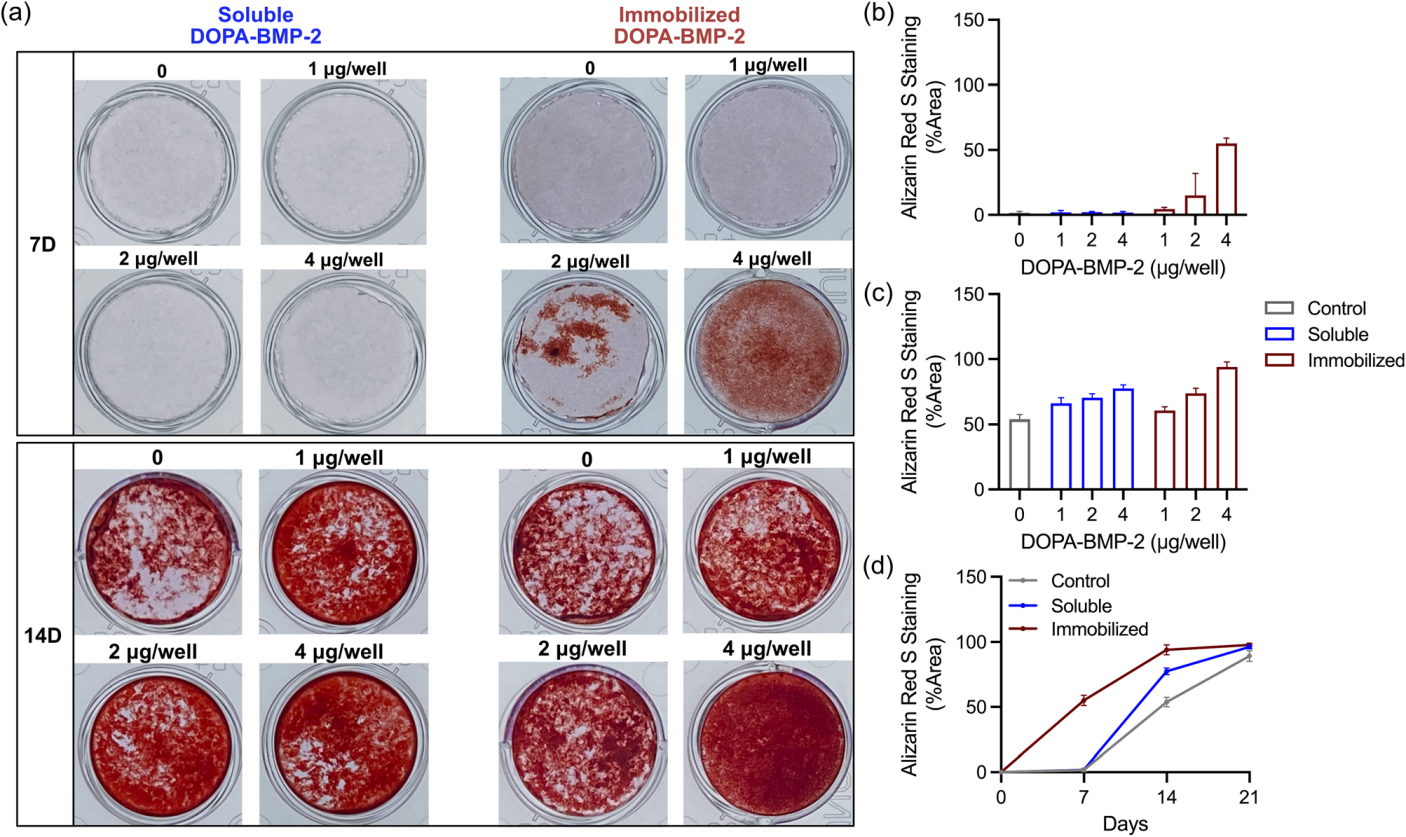
(a) Representative images of mineralized MC3T3-E1 cells cultured on immobilized DOPA-BMP-2 surfaces or with soluble DOPA-BMP-2 for 7 and 14 days. The cells are stained with Alizarin Red S, and red stains indicate calcium deposition in the cells. Percentage of calcification coverage of MC3T3-E1 cultured on immobilized DOPA-BMP-2 surfaces or soluble DOPA-BMP-2 for (b) 7 and (c) 14 days. (d) Time-course analysis of mineralization in the control, soluble, and immobilized DOPA-BMP-2 groups (4 μg per well).

The long-term effects of growth factor-functionalized surfaces were assessed by measuring calcium deposition, an indicator of late-stage osteogenic differentiation. Fig. 4 presents representative Alizarin Red S staining images and quantitative analysis of mineral deposition in response to various concentrations of soluble or immobilized DOPA-BMP-2 at different time points. On day 7, calcium deposition was not detected in the control (0 μg per well) or soluble DOPA-BMP-2 groups. In contrast, the immobilized DOPA-BMP-2 group exhibited clear calcium deposition at 2 and 4 μg per well conditions, with 15.1% and 55.1% mineralized area, respectively, indicating a stronger and earlier osteogenic response (Fig. 4a and b). On day 14, both the soluble and immobilized DOPA-BMP-2 groups demonstrated concentration-dependent increases in mineralization. The immobilized group at 4 μg per well showed more mineral deposition (93.9%) than the soluble group (77.5%) (Fig. 4a and c). On day 21, although all conditions exhibited extensive mineralization with only modest differences across groups, immobilized DOPA-BMP-2 showed a slightly higher stained area than its soluble counterpart, such as at concentrations of 2 and 4 μg per well, suggesting a more efficient mineralization process (Fig. S2, ESI†).

To further demonstrate the progression of mineralization, Fig. 4d displays a time-course analysis for the control, soluble, and immobilized DOPA-BMP-2 groups at 4 μg per well (days 7, 14, and 21). Mineralization progressed most rapidly in the immobilized group, followed by that in the soluble group, with the control group showing the slowest progression. The immobilized group reached a plateau by Day 14 and exhibited 97.8% mineralization on Day 21. In contrast, mineralization in the soluble and control groups continued to increase throughout the 21 days period, ultimately reaching 96.1% and 89.2%, respectively.

The enhanced biological activity of immobilized DOPA-BMP-2 is likely attributable to the distinct spatial presentation of BMP-2 for cells. Unlike the three-dimensional distribution of soluble BMP-2, immobilized BMP-2 was present in a two-dimensional manner, creating a high local concentration of BMP-2 near the ventral cell membrane. This increases the availability of BMP-2 to its receptors, amplifying downstream signaling pathways such as Smad-dependent and mitogen-activated protein kinase/extracellular signal-regulated kinase (MAPK/ERK) signaling.^36–38^ Additionally, immobilization restricts the internalization of ligand-receptor complexes, contributing to sustained signaling effects.^39^ These findings confirmed that DOPA-BMP-2-immobilized surfaces exhibit high bioactivity and osteoinductive potential, offering a promising approach to enhance osteogenic differentiation.

### 3.3 Combined effect of BMP-2 immobilization and ultrasound on osteogenesis

To determine the appropriate ultrasound conditions for osteogenesis studies, the biocompatibility of LIPUS with the MC3T3-E1 cell line was assessed through cell growth measurements (Fig. S3, ESI†). Ultrasound at 30 or 60 mW cm^−2^ was applied for 20 or 60 min per day. Cell growth increased to approximately 108% at 30 mW cm^−2^ for 20 min per day and further to 119% when the treatment period was extended to 60 min. At 60 mW cm^−2^, preosteoblast growth was stimulated to 119% with 20 minute treatments but decreased to 88% with prolonged exposure (60 min per day). The enhanced growth observed with LIPUS treatment was likely because of Rho-associated protein kinase-mediated ERK1/2 phosphorylation, as previously reported for this cell line.^40,41^ However, mechanical disruption caused by prolonged exposure to higher doses at 60 mW cm^−2^ may hinder cell proliferation. Considering the reciprocal inhibition of proliferation and differentiation during skeletal repair that is mediated by Notch-Wnt signaling switches,^42^ 30 mW cm^−2^ for 20 min per day—which moderately induced proliferation—was selected for further osteogenic studies.

**Fig. 5:**
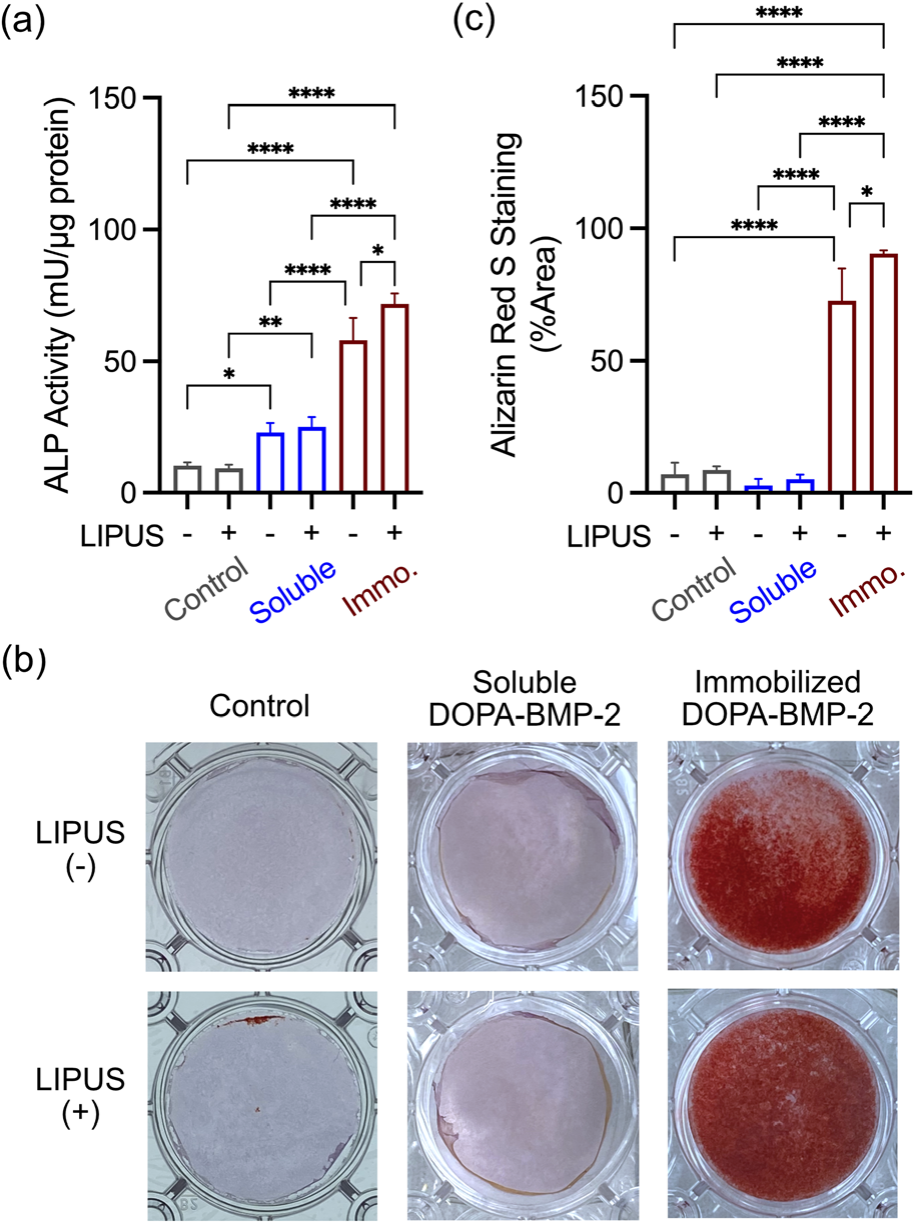
(a) ALP activity of MC3T3-E1 cells after 3 days culture, (b) representative figures of calcium deposition on day 7, and (c) percentage of calcium deposition coverage with combinational treatment of ultrasound and DOPA-BMP-2 of 4 μg per well. The condition, 30 mW cm^−2^ of intensity and 20 min per day is applied. Cells are stained by Alizarin Red S, and red stains indicate calcium deposition of cells. *p < 0.05, **p < 0.01, and ****p < 0.0001.

To evaluate the combined effect of ultrasound and DOPA-BMP-2 in immobilized or soluble form, early- and late-stage differentiation of MC3T3-E1 cells was investigated. As shown in Fig. 5a, early-stage differentiation was assessed on day 4. In the control and soluble DOPA-BMP-2 groups, ultrasound had a limited effect on ALP expression, with comparable levels observed between ultrasound-treated and untreated conditions. In contrast, immobilized DOPA-BMP-2 significantly enhanced ALP activity. The combination of immobilized DOPA-BMP-2 and ultrasound further enhanced ALP expression, increasing the activity from 58.1 to 71.1 mU μg_protein_^−1^.

The combined effect of LIPUS and BMP-2-immobilized surfaces was further assessed by analyzing mineralization after 7 days of treatment (Fig. 5b and c). Consistent with the ALP activity results, mineral calcium deposition was not observed in the ultrasound-treated control group or in the group treated with a combination of soluble DOPA-BMP-2 and ultrasound. However, a significant enhancement was evident with the combined treatment of LIPUS and immobilized DOPA-BMP-2, resulting in a mineralized area of 90.4%, compared to 72.6% without ultrasound.

The results revealed an intriguing phenomenon—although LIPUS alone did not induce differentiation in preosteoblasts, osteogenic differentiation was significantly enhanced by ultrasound when BMP-2 was immobilized on the substrate surface. In contrast, the combination of soluble BMP-2 and ultrasound did not achieve this enhanced effect. These findings highlight the crucial role of BMP-2 spatial presentation at the cell–substrate interface in facilitating the interaction between biophysical and biochemical cues for osteogenesis.

### 3.4 Ultrasound-stimulated mechanosensing

**Fig. 6:**
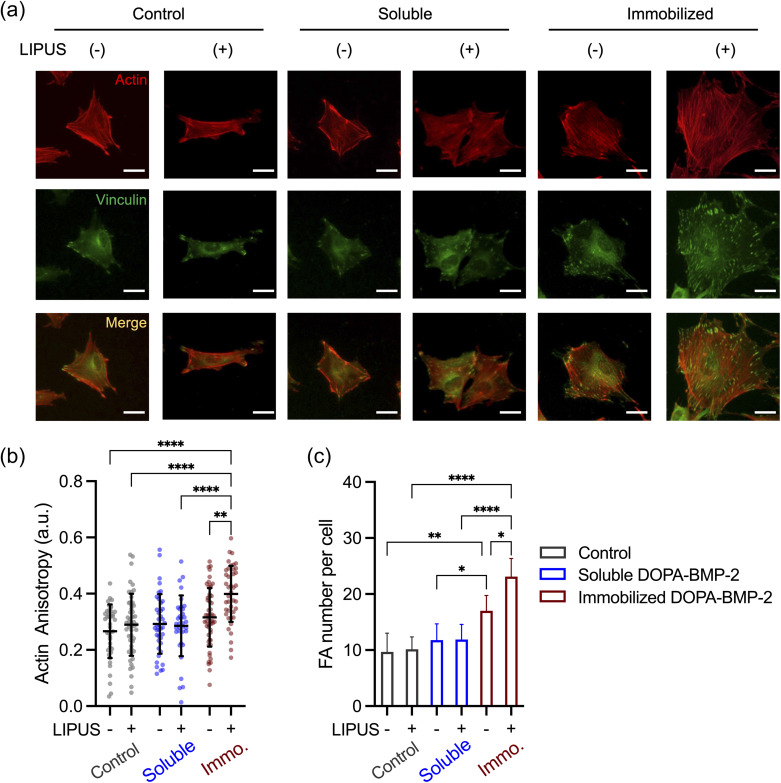
(a) Representative fluorescent images of preosteoblasts exposed to combined treatment with DOPA-BMP-2 and ultrasound. Red: F-actin. Green: vinculin. The two channels are merged in the third row. Scale bar: 50 μm. (b) Quantification of actin anisotropy. (c) Average FA number in each cell. *p < 0.05, **p < 0.01, and ****p < 0.0001.

To assess how ultrasound affects subcellular structures during combined treatment with BMP-2, staining of vinculin and actin filaments was performed (Fig. 6a). Compared with untreated cells in the control group (ground state), cells treated with LIPUS, immobilized DOPA-BMP-2, or soluble DOPA-BMP-2 alone demonstrated no significant alterations in cytoskeletal organization, as indicated by actin anisotropy, which reflects the degree of actin alignment and organization (Fig. 6a and b). When LIPUS was combined with immobilized DOPA-BMP-2, the cells exhibited significantly structured actin cytoskeleton and the highest actin anisotropy, reaching 0.40, compared to 0.32 in the immobilized groups without ultrasound (Fig. 6b). While focal adhesion (FA) formation was unaffected by soluble BMP-2 alone or in combination with ultrasound, immobilized DOPA-BMP-2 significantly enhanced FA assembly, increasing the average FA number per cell to 17.0, and its combination with LIPUS further induced the FA assembly to 23.1 FAs per cell (Fig. 6c).

FA links integrins to the cytoskeleton and is crucial for transmitting extracellular mechanical signals to the cytoplasm and nucleus, thereby regulating downstream transcriptional responses.^43,44^ This enhancement of cytoskeleton remodeling and FA assembly is attributable to BMP-2 presentation at the cell–substrate interface and the potential interactions between BMP receptors and integrins when ultrasound waves served as mechanical inputs.^45^

**Fig. 7:**
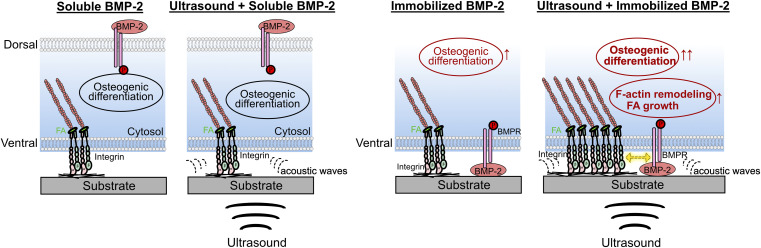
Summary of the combined effect of biophysical ultrasound and DOPA-BMP-2 in soluble or immobilized form.

The observed phenomena are summarized in Fig. 7. ALP expression and mineralization results confirmed the superior osteoinductive properties of DOPA-BMP-2-modified surfaces, supporting the effectiveness of the recombinant BMP-2 variant and surface modification strategy. Osteogenic induction is driven by BMP-2 interactions with its transmembrane heterodimeric receptors (BMPRI and BMPRII) and the activation of downstream signaling pathways. The combination of LIPUS and soluble DOPA-BMP-2 did not significantly alter subcellular structures or enhance differentiation. In contrast, when immobilized DOPA-BMP-2 was presented to the ventral cell surface, ultrasound stimulated FA formation and cytoskeleton remodeling, indicating that the acoustic force activated integrin-associated FAs in the presence of BMP receptors, thereby amplifying osteogenic differentiation. These results align with previous studies that reported no synergistic effect on the osteogenesis of bone marrow stromal cells when LIPUS was combined with soluble BMP-2, emphasizing the significance of BMP-2 spatial presentation.^19^ Notably, BMPRII has been identified as a mechanoreceptor in endothelial cells, mediating sustained BMPRI-αvβ3 integrin associations under oscillatory shear forces.^46^ It is possible that BMPR in preosteoblasts plays a role in mechanotransduction and mediates integrin-associated pathways.

In summary, these findings indicate that in preosteoblasts, BMP receptors on the ventral cell membrane may function as mechanosensory counterparts, cooperating with integrins to respond to external acoustic forces. This interaction facilitates the transduction of mechanical stimuli through FAs and the cytoskeleton, thereby regulating transcriptional programs for osteogenesis.

## 4 Conclusions

This study demonstrated the significant osteoinductive potential of DOPA-BMP-2 immobilized on substrate surfaces and its additional effects when combined with LIPUS. Immobilized DOPA-BMP-2 exhibited superior biological activity compared to its soluble form, as shown by the increased ALP activity and accelerated mineralization (93.9% *vs.* 77.5% on day 14). Ultrasound further facilitated cytoskeleton remodeling, FA formation, and osteogenic differentiation, but only when combined with immobilized DOPA-BMP-2, resulting in increased ALP expression (71.7 *vs.* 58.1 mU μg_protein_^−1^) and mineralized area from 72.6% to 90.4% on Day 7. These results highlight the significance of spatial presentation of growth factors in enhancing mechanotransduction and cellular functions.

## Author contributions

K. Fang: conceptualization, methodology, investigation, data curation, formal analysis, visualization, writing – original draft preparation; M. Ueda: supervision, validation, writing – reviewing & editing; X. Ren: methodology, investigation; Y. Nakagawa: validation; Y. Anraku: validation; T. Ikoma: supervision; Y. Ito: conceptualization, writing – reviewing & editing, supervision.

## Conflicts of interest

There are no conflicts to declare.

## Supplementary Material

RA-015-D5RA02354H-s001

RA-015-D5RA02354H-s002

RA-015-D5RA02354H-s003

## Data Availability

The data supporting this article have been included as part of the ESI.†

## References

[cit1] Salazar V. S., Gamer L. W., Rosen V. (2016). Nat. Rev. Endocrinol..

[cit2] Carragee E. J., Hurwitz E. L., Weiner B. K. (2011). Spine J..

[cit3] Gilde F., Fourel L., Guillot R., Pignot-Paintrand I., Okada T., Fitzpatrick V., Boudou T., Albiges-Rizo C., Picart C. (2016). Acta Biomater..

[cit4] Ziegler J., Anger D., Krummenauer F., Breitig D., Fickert S., Guenther K. P. (2008). J. Biomed. Mater. Res..

[cit5] Masters K. S. (2011). Macromol. Biosci..

[cit6] Siverino C., Fahmy-Garcia S., Mumcuoglu D., Oberwinkler H., Muehlemann M., Mueller T., Farrell E., van Osch G., Nickel J. (2022). Int. J. Mol. Sci..

[cit7] Hettiaratchi M. H., Krishnan L., Rouse T., Chou C., McDevitt T. C., Guldberg R. E. (2020). Sci. Adv..

[cit8] Sarrigiannidis S. O., Dobre O., Navarro A. R., Dalby M. J., Gonzalez-Garcia C., Salmeron-Sanchez M. (2023). Mater. Today Bio.

[cit9] Llopis-Hernandez V., Cantini M., Gonzalez-Garcia C., Cheng Z. A., Yang J., Tsimbouri P. M., Garcia A. J., Dalby M. J., Salmeron-Sanchez M. (2016). Sci. Adv..

[cit10] Lee H., Dellatore S. M., Miller W. M., Messersmith P. B. (2007). Science.

[cit11] Ryu J. H., Messersmith P. B., Lee H. (2018). ACS Appl. Mater. Interfaces.

[cit12] Tada S., Ren X., Mao H., Heo Y., Park S. H., Isoshima T., Zhu L., Zhou X., Ito R., Kurata S., Osaki M., Kobatake E., Ito Y. (2021). Adv. Sci..

[cit13] Ren X., Tsuji H., Uchino T., Kono I., Isoshima T., Okamoto A., Nagaoka N., Ozaki T., Matsukawa A., Miyatake H., Ito Y. (2024). J. Mater. Chem. B.

[cit14] Zhang C., Miyatake H., Wang Y., Inaba T., Wang Y., Zhang P., Ito Y. (2016). Angew Chem. Int. Ed. Engl..

[cit15] Heckman J. D., Ryaby J. P., McCabe J., Frey J. J., Kilcoyne R. F. (1994). J. Bone Jt. Surg., Am. Vol..

[cit16] Gebauer D., Mayr E., Orthner E., Ryaby J. P. (2005). Ultrasound Med. Biol..

[cit17] Guo X., Lv M., Lin J., Guo J., Lin J., Li S., Sun Y., Zhang X. (2024). J. Ultrasound Med..

[cit18] Harrison A., Lin S., Pounder N., Mikuni-Takagaki Y. (2016). Ultrasonics.

[cit19] Sant'Anna E. F., Leven R. M., Virdi A. S., Sumner D. R. (2005). J. Orthop. Res..

[cit20] Lai C. H., Chen S. C., Chiu L. H., Yang C. B., Tsai Y. H., Zuo C. S., Chang W. H., Lai W. F. (2010). Ultrasound Med. Biol..

[cit21] Zhu H., Shi Z., Cai X., Yang X., Zhou C. (2020). Exp. Ther. Med..

[cit22] Han J. J., Yang H. J., Hwang S. J. (2022). Tissue Eng. Regener. Med..

[cit23] Wang Y. K., Yu X., Cohen D. M., Wozniak M. A., Yang M. T., Gao L., Eyckmans J., Chen C. S. (2012). Stem Cells Dev..

[cit24] Brunner M., Mandier N., Gautier T., Chevalier G., Ribba A. S., Guardiola P., Block M. R., Bouvard D. (2018). PLoS One.

[cit25] Wei Q., Holle A., Li J., Posa F., Biagioni F., Croci O., Benk A. S., Young J., Noureddine F., Deng J., Zhang M., Inman G. J., Spatz J. P., Campaner S., Cavalcanti-Adam E. A. (2020). Adv. Sci..

[cit26] Patntirapong S., Chanruangvanit C., Lavanrattanakul K., Satravaha Y. (2021). Acta Histochem..

[cit27] Weng S. N., Shao Y., Chen W. Q., Fu J. P. (2016). Nat. Mater..

[cit28] Horzum U., Ozdil B., Pesen-Okvur D. (2014). MethodsX.

[cit29] Posa F., Baha-Schwab E. H., Wei Q., Di Benedetto A., Neubauer S., Reichart F., Kessler H., Spatz J. P., Albiges-Rizo C., Mori G., Cavalcanti-Adam E. A. (2021). Biomaterials.

[cit30] Boudaoud A., Burian A., Borowska-Wykret D., Uyttewaal M., Wrzalik R., Kwiatkowska D., Hamant O. (2014). Nat. Protoc..

[cit31] Fraioli R., Neubauer S., Rechenmacher F., Bosch B. M., Dashnyam K., Kim J. H., Perez R. A., Kim H. W., Gil F. J., Ginebra M. P., Manero J. M., Kessler H., Mas-Moruno C. (2019). Biomater. Sci..

[cit32] Migliorini E., Thakar D., Sadir R., Pleiner T., Baleux F., Lortat-Jacob H., Coche-Guerente L., Richter R. P. (2014). Biomaterials.

[cit33] Li Y. R., Qin M., Li Y., Cao Y., Wang W. (2014). Langmuir.

[cit34] Li M., Xi N., Wang Y. C., Liu L. Q. (2019). ACS Biomater. Sci. Eng..

[cit35] Gelebart P., Cuenot S., Sinquin C., Halgand B., Sourice S., Le Visage C., Guicheux J., Colliec-Jouault S., Zykwinska A. (2022). Carbohydr. Polym..

[cit36] Crouzier T., Fourel L., Boudou T., Albiges-Rizo C., Picart C. (2011). Adv. Mater..

[cit37] Massague J., Seoane J., Wotton D. (2005). Genes Dev..

[cit38] Nohe A., Hassel S., Ehrlich M., Neubauer F., Sebald W., Henis Y. I., Knaus P. (2002). J. Biol. Chem..

[cit39] Schwab E. H., Pohl T. L., Haraszti T., Schwaerzer G. K., Hiepen C., Spatz J. P., Knaus P., Cavalcanti-Adam E. A. (2015). Nano Lett..

[cit40] Kusuyama J., Bandow K., Shamoto M., Kakimoto K., Ohnishi T., Matsuguchi T. (2014). J. Biol. Chem..

[cit41] Zhang G., Li X., Wu L., Qin Y. X. (2021). Bone Res..

[cit42] Lee S., Remark L. H., Josephson A. M., Leclerc K., Lopez E. M., Kirby D. J., Mehta D., Litwa H. P., Wong M. Z., Shin S. Y., Leucht P. (2021). npj Regener. Med..

[cit43] Geiger B., Yamada K. M. (2011). Cold Spring Harb Perspect Biol..

[cit44] Ringer P., Colo G., Fassler R., Grashoff C. (2017). Matrix Biol..

[cit45] Fourel L., Valat A., Faurobert E., Guillot R., Bourrin-Reynard I., Ren K., Lafanechere L., Planus E., Picart C., Albiges-Rizo C. (2016). J. Cell Biol..

[cit46] Zhou J., Lee P. L., Lee C. I., Wei S. Y., Lim S. H., Lin T. E., Chien S., Chiu J. J. (2013). J. Thromb. Haemostasis.

