# Supplementary material for: Osteogenesis enhancement by immobilized DOPA-BMP-2 in combination with ultrasonic stimulation
Source: RSC Adv. 2025 Jun 11;15(25):19860–9. doi: 10.1039/d5ra02354h (PMC12152856; doi:10.1039/d5ra02354h)
Supplement: RA-015-D5RA02354H-s001 [file RA-015-D5RA02354H-s001.pdf]

Supplementary Information (SI) for RSC Advances.
This journal is © The Royal Society of Chemistry 2025

Supporting Information

Osteogenesis enhancement by immobilized DOPA-BMP-2 in combination with ultrasonic stimulation

Kun Fang, [a, b] *Motoki Ueda,* [a, c] *Xueli Ren,* [a] *Yasuhiro Nakagawa,* [b] *Yasutaka Anraku,* [b] *Toshiyuki Ikoma,* [b] *and Yoshihiro Ito*[*, a, b, c]

a. Nano Medical Engineering Laboratory, RIKEN Cluster for Pioneering Research, Saitama 351-0198 Japan.

b. Graduate School of Material Science and Engineering, Institute of Science Tokyo, Tokyo 152-8550, Japan.

c. Emergent Bioengineering Materials Research Team, RIKEN Center for Emergent Matter Science, Saitama, 351-0198, Japan.

* Corresponding author, Email: y-ito@riken.jp

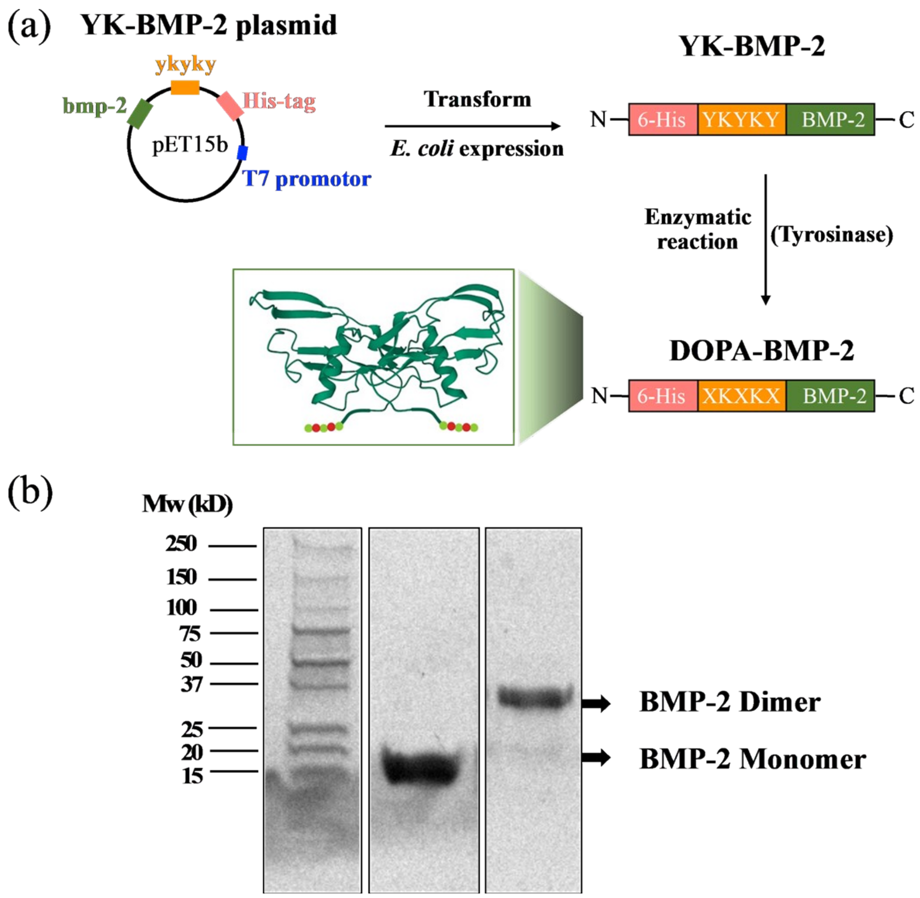

Fig. S1. (a) Recombinant plasmids of YK-BMP-2 and DOPA-BMP-2 preparation. Y: tyrosine, K: lysine. (b) SDS-PAGE results for purified recombinant BMP-2 monomers and dimers.

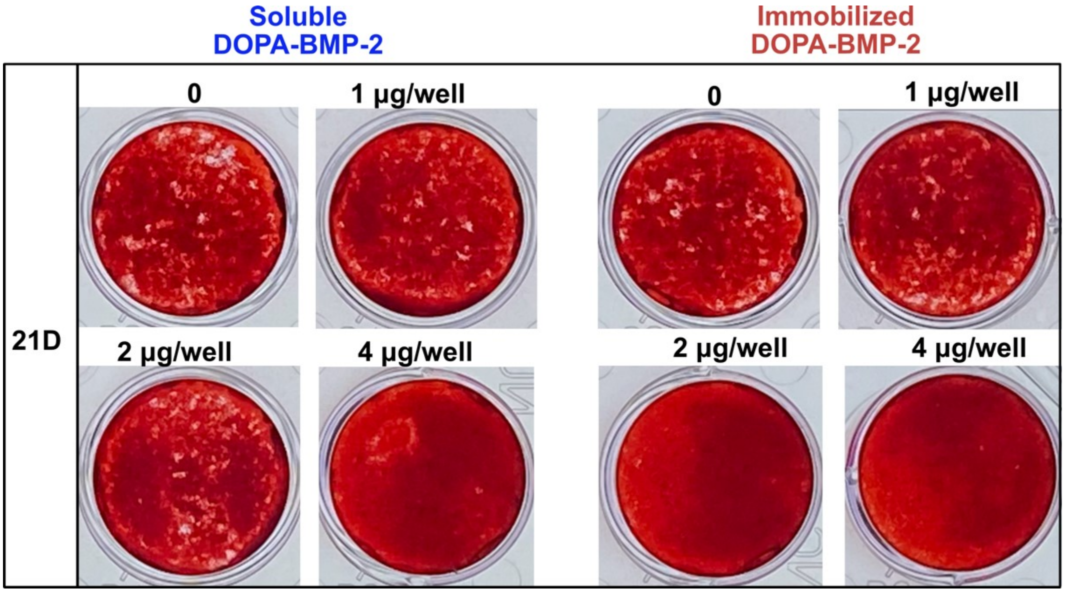

Fig. S2. Representative alizarin red S staining images of MC3T3-E1 cells cultured for 21 days on immobilized DOPA-BMP-2 surfaces or with soluble DOPA-BMP-2 at 1, 2, and 4 μg/well.

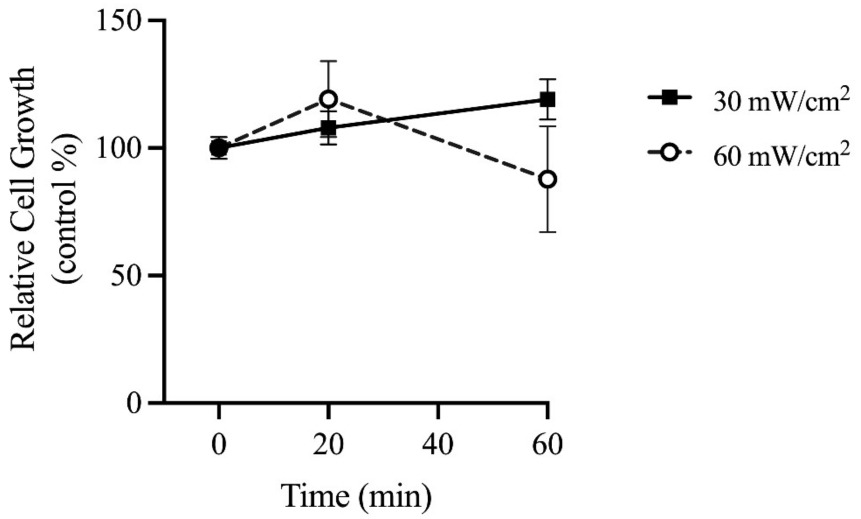

Fig. S3. Cell growth of MC3T3-E1 cells treated with LIPUS for 20 or 60 min per day, and the intensity of the ultrasound is either 30 or 60 mW/cm^2.
