# Supplementary material for: Osteogenesis enhancement by immobilized DOPA-BMP-2 in combination with ultrasonic stimulation
Source: RSC Adv. 2025 Jun 11;15(25):19860–9. doi: 10.1039/d5ra02354h (PMC12152856; doi:10.1039/d5ra02354h)
Supplement: RA-015-D5RA02354H-s002 [file RA-015-D5RA02354H-s002.pdf]

Supplementary Information (SI) for RSC Advances.
This journal is © The Royal Society of Chemistry 2025

Full gel electrophoresis image of Figure S1(b)

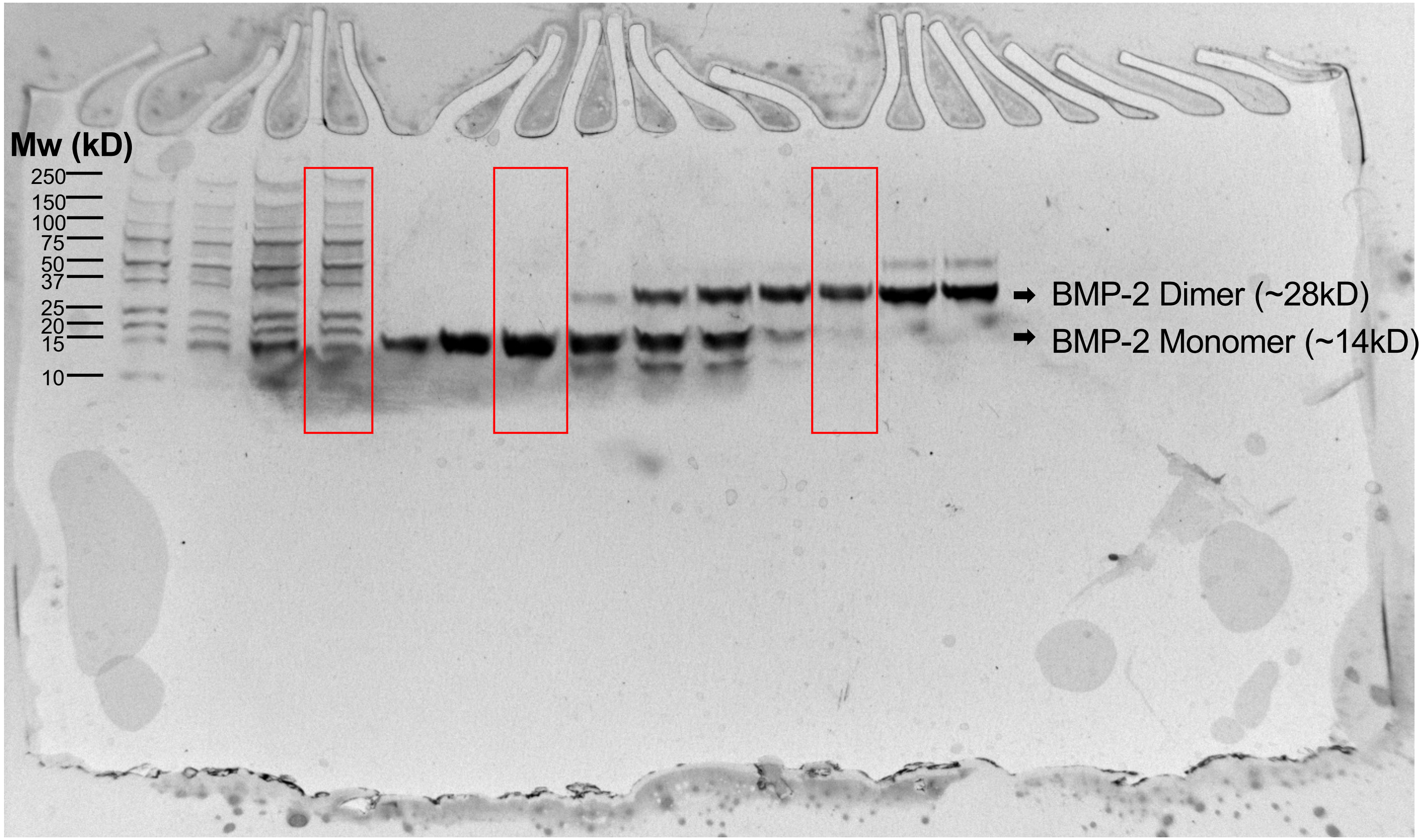

Results in red frames were presented in Figure S1(b) to indicate BMP-2 monomer and dimer, respectively.
