# Supplementary material for: Osteogenesis enhancement by immobilized DOPA-BMP-2 in combination with ultrasonic stimulation
Source: RSC Adv. 2025 Jun 11;15(25):19860–9. doi: 10.1039/d5ra02354h (PMC12152856; doi:10.1039/d5ra02354h)
Supplement: RA-015-D5RA02354H-s003 [file RA-015-D5RA02354H-s003.pdf]

This journal is © The Royal Society of Chemistry 2025

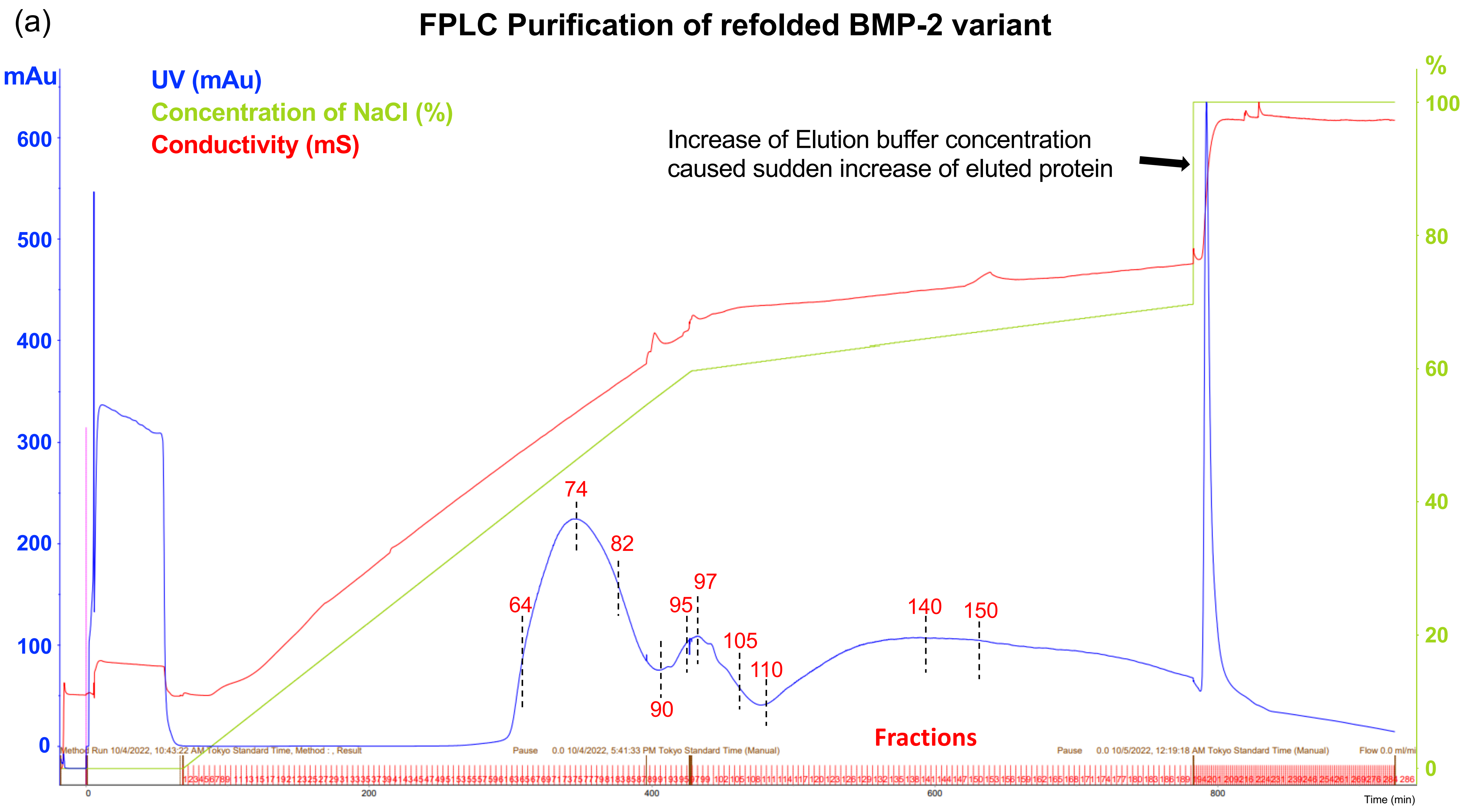

(b)

Full gel electrophoresis image of Figure S1(b)

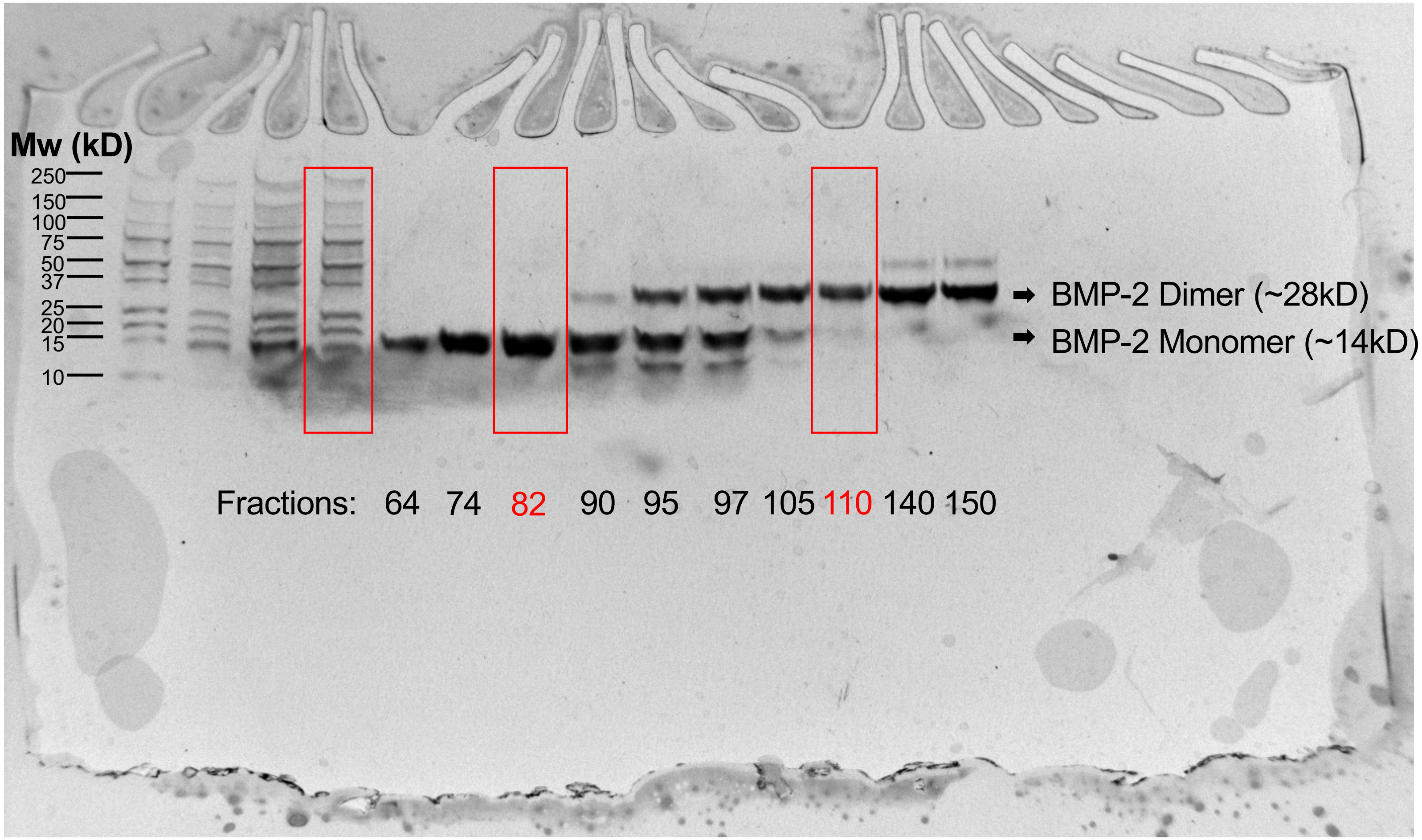

Supplement Data for BMP-2 purification and gel electrophoresis

Figure (a) Purification of refolded BMP-2 homodimer by FPLC using heparin column, and **(b)** Non-reduced SDS-page full image of refolded BMP-2 in different fractions. Results in red frames were presented in Figure S1(b) to indicate BMP-2 monomer and dimer, respectively.
